# Staged Single Ventricle Palliation with Pulmonary Artery Rehabilitation for Unguarded Tricuspid Orifice and Hypoplastic Left Pulmonary Artery

**DOI:** 10.1093/icvts/ivag027

**Published:** 2026-01-25

**Authors:** Hiroshi Manome, Takaya Hoashi, Koichi Toda, Takaaki Suzuki

**Affiliations:** Department of Pediatric Cardiovascular Surgery, International Medical Center, Saitama Medical University, Saitama 350-1298, Japan; Department of Pediatric Cardiovascular Surgery, International Medical Center, Saitama Medical University, Saitama 350-1298, Japan; Department of Pediatric Cardiology, International Medical Center, Saitama Medical University, Saitama 350-1298, Japan; Department of Pediatric Cardiovascular Surgery, International Medical Center, Saitama Medical University, Saitama 350-1298, Japan

**Keywords:** UGTO, single ventricular palliation, pulmonary artery rehabilitation

## Abstract

The patient was diagnosed with unguarded tricuspid orifice (UGTO), functional pulmonary atresia, and left pulmonary artery hypoplasia. In view of the severe right ventricular dysfunction, the staged single ventricular palliation procedure was selected. The surgical procedure on Starnes operation and left pulmonary artery augmentation was performed one month after birth. As left pulmonary artery stenosis was diagnosed, secondly, the bidirectional cavopulmonary shunt with additional systemic to pulmonary shunt and intrapulmonary patch septation and left pulmonary artery augmentation with in-situ pericardium were performed. Despite the necessity for additional balloon dilation and surgical blunt dilation, the total cavopulmonary connection operation was ultimately performed, resulting in the successful implementation of staged single-ventricle palliation in conjunction with left pulmonary artery rehabilitation.

## INTRODUCTION

Unguarded tricuspid orifice (UGTO) constitutes a rare congenital cardiac malformation, characterized by the absence of tricuspid valve (TV) leaflets and a normal annulus. It has been associated with pulmonary atresia and right ventricular dysfunction, in a manner analogous to Ebstein’s anomaly with pulmonary atresia or pulmonary atresia with an intact ventricular septum.^1^ Consequently, the approach of single ventricular palliation or TV replacement is determined by the severity of right ventricular dysfunction.^2^^,^^3^ In this study, we present a case of a patient with UGTO, functional pulmonary atresia, and hypoplastic left pulmonary artery who was successfully treated with staged single-ventricle palliation associated with left pulmonary artery rehabilitation.

## CASE

**Figure 1.**
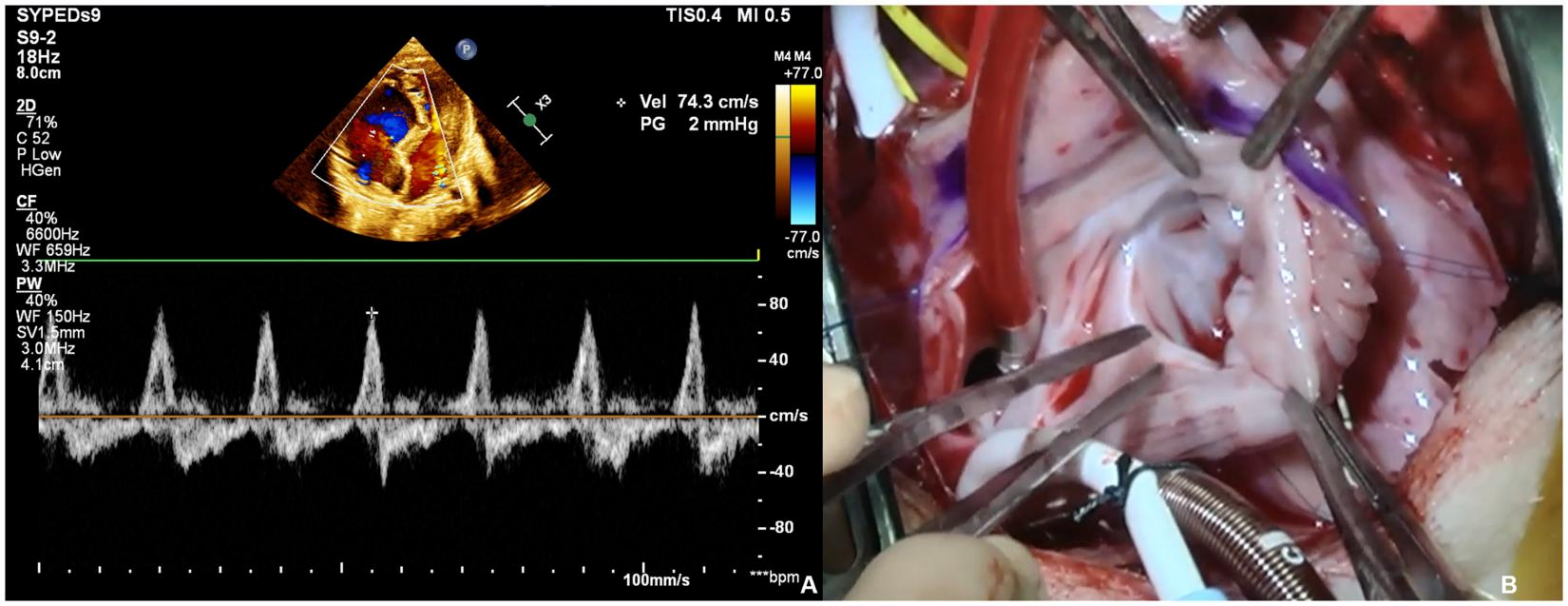
(A) Pulsed Doppler Image of Trans-Thoracic Echocardiogram Showed Free Regurgitation on the Tricuspid Valve Position and (B) Intraoperative Inspections of UGTO.

The presence of fetal echocardiographic findings suggested the possibility of pulmonary atresia with intact ventricular septum. The infant was delivered at 39 weeks’ gestation, with a birth weight of 3452 g. The administration of prostaglandin E1 was initiated. Trans-thoracic echocardiogram revealed UGTO with free tricuspid insufficiency (Video 1, Fig. 1A). The left ventricle was compressed by the dilated right ventricle, and the left ventricular side of the intraventricular septum appeared non-compaction. The arterial duct was found to drain into the left pulmonary artery, and the left pulmonary artery beyond the ductal insertion was found to be hypoplastic.

The Starnes operation was performed one month after birth as the first palliative surgery (Video 2). The UGTO was closed in part with an expanded polytetrafluoroethylene (ePTFE) patch, and the left pulmonary artery was augmented with an ePTFE patch.

**Figure 2.**
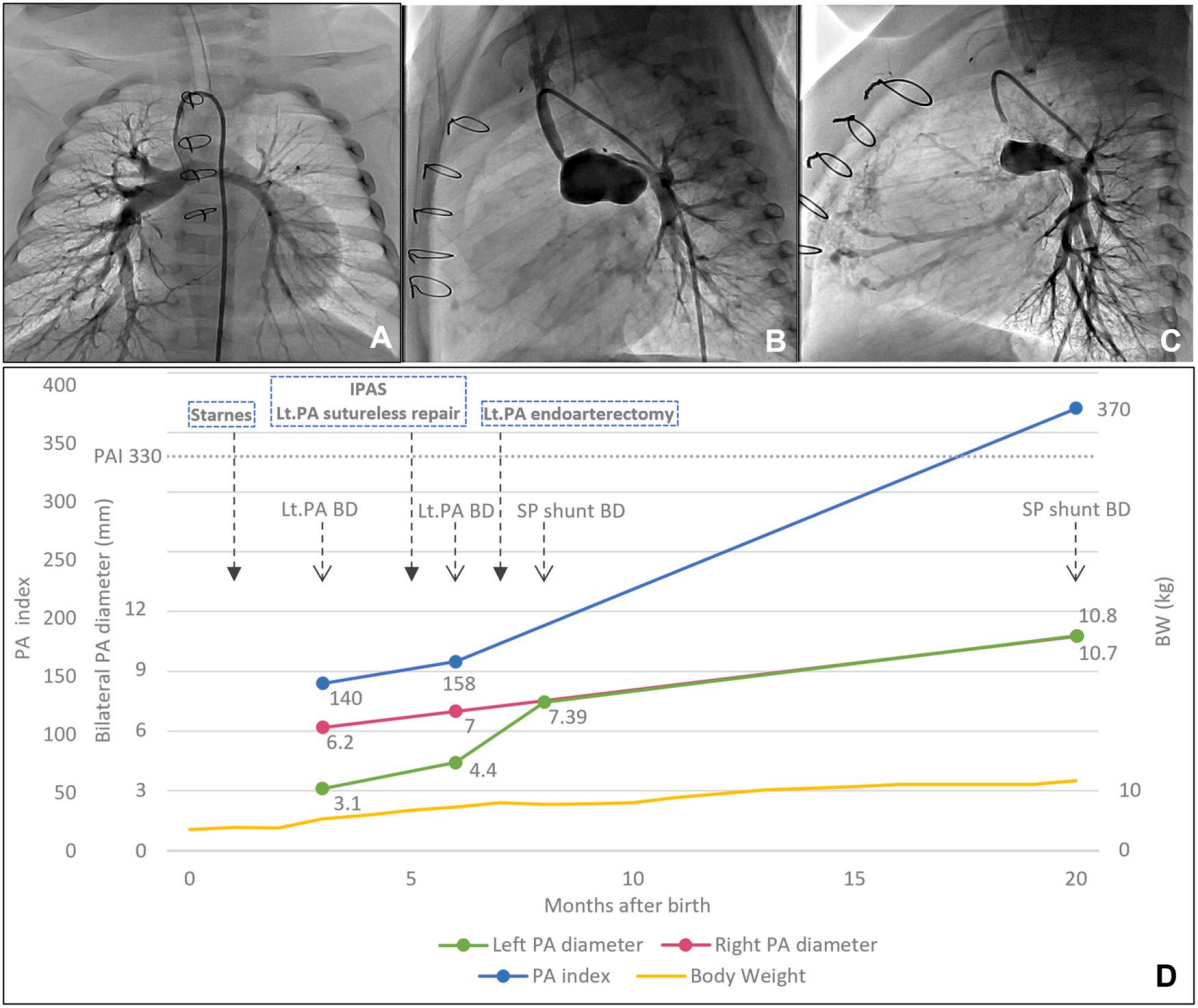
Pulmonary Angiography Findings of (A) after the Starnes Operation, (B) after the Intrapulmonary Artery Septation with Suture-Less Left Pulmonary Artery Augmentation, (C) after the Additional Pulmonary Artery Augmentation, (D) Serial Changes of Pulmonary Artery Size Represented by Pulmonary Artery Index (Nakata Index).

The pulmonary angiogram 2 months after the Starnes procedure revealed residual left pulmonary artery stenosis (Fig. 2A). The procedure of catheter-based balloon dilation was undertaken, yet no improvement was observed. The second operation consisted of bidirectional cavopulmonary shunt with additional systemic to pulmonary shunt and intrapulmonary artery septation with an ePTFE patch, and the redo left pulmonary artery augmentation with in-situ pericardium using suture-less technique (Video 3).^4^^,^^5^ A planned percutaneous balloon pulmonary angioplasty was attempted one month after the second operation; however, due to a significant change in caliber, it was impossible to insert the guidewire into the distal left pulmonary artery (Fig. 2B). Therefore, the distal left pulmonary artery cutback and blunt dilation were performed surgically. The subsequent findings included confirmation of growth of the distal left pulmonary artery (Fig. 2C).

Pre-Fontan evaluation was performed at 20 months of age, revealing equally developed pulmonary arteries on both sides. At that point, pulmonary artery index: Nakata Index was 330, Mcgoon ratio was 2.42, and lower lobe index was 204. Total cavopulmonary connection was achieved using an 18-mm-diameter ePTFE extra-cardiac conduit with a 3.5-mm fenestration.

## COMMENT

Two case reports of successful single ventricular palliation for this extremely rare cardiac malformation presented different surgical options with or without right ventricular exclusion.^3^^,^^4^ In the presented case, associated functional pulmonary atresia indicated right ventricular dysfunction, and dilated right ventricle compressed the left ventricle. Additionally, the left side of the ventricular septum initially exhibited non-compaction-like changes. Therefore, right ventricular exclusion was chosen as the initial palliation to preserve left ventricular function.

Combination of intrapulmonary artery septation^4^ and suture-less left pulmonary artery augmentation^5^ facilitated the development of the left pulmonary artery. Unfortunately, the planned catheter-based dilation of the remaining distal pulmonary artery stenosis beyond the pericardial reflex was unsuccessful due to the significant diameter difference between the enlarged proximal artery and the remaining narrow distal artery. It was hypothesized that pulmonary artery cutback beyond the pericardial reflex and dilation from the inside, which was consequently performed as an additional surgery in the presented case, should have been performed concomitantly with suture-less repair.

In conclusion, a patient with UGTO, functional pulmonary atresia, and hypoplastic left pulmonary artery was successfully treated with staged single ventricular palliation concomitant with surgical left pulmonary artery rehabilitation.

## Data Availability

The data underlying this article are available in the article.

## References

[1] Anderson RH, Silverman NH, Zuberbuhler JR. Congenitally unguarded tricuspid orifice: its differentiation from Ebstein’s malformation in association with pulmonary atresia and intact ventricular septum. Pediatr Cardiol. 1990;11:86-90. 2349148 10.1007/BF02239568

[2] Kariya T, Imai Y, Murakami A, et al Images in cardiovascular medicine. Markedly dilated right heart 17 years after initial treatment repaired by total right ventricular exclusion and total cavopulmonary connection. Circulation. 2008;118:e133-e135 18725496 10.1161/CIRCULATIONAHA.107.760561

[3] Hirota M, Kawada M, Ishino K, et al Staged Fontan’s operation for unguarded tricuspid orifice with pulmonary atresia. Eur J Cardiothorac Surg. 2008;34:1111-1112. 18760620 10.1016/j.ejcts.2008.07.054

[4] Sakamoto K, Ikai A, Fujimoto Y, et al Novel surgical approach ‘intrapulmonary-artery septation’ for Fontan candidates with unilateral pulmonary arterial hypoplasia or pulmonary venous obstruction. Interact CardioVasc Thorac Surg. 2007;6:150-154. 17669796 10.1510/icvts.2005.124925

[5] Juaneda I, Peirone A, Diaz J, et al Repair of the left pulmonary artery after bilateral banding in hybrid procedures: results using “sutureless” repair technique. World J Pediatr Congenit Heart Surg. 2016;7:89-92. 26714999 10.1177/2150135115606623

